# The value of serum CA125 for the development of virtual follow-up strategies for patients with epithelial ovarian cancer: a retrospective study

**DOI:** 10.1186/1757-2215-5-11

**Published:** 2012-03-22

**Authors:** Elizabeth Varughese, Srinivas Kondalsamy-Chennakesavan, Andreas Obermair

**Affiliations:** 1 Greenslopes Private Hospital, Greenslopes, Brisbane, Queensland, Australia; 2 The University of Queensland, School of Medicine, Brisbane, Queensland, Australia

**Keywords:** Ovarian cancer, CA-125, Recurrence, Diagnosis, Follow-up

## Abstract

**Background:**

Serum CA125 is routinely used in the follow up of ovarian cancer. The objective of the present study was to evaluate the usefulness of CA125 in the detection of ovarian cancer recurrence.

**Methods:**

This retrospective case study was carried out at a tertiary gynaecological cancer centre in Australia. Patients with all cell types of epithelial ovarian cancer (EOC) treated between 2003 and2010 were considered eligible. We excluded patients whose aim of treatment was palliative, had no follow-up, had no pre-operative CA125 reading or had pre-operative CA125 levels < 35 U/mL. After primary treatment, patients were followed up as per guidelines suggested by National Comprehensive Cancer Network (NCCN). We recorded if symptoms, findings from physical examination, imaging or serum CA125 levels led to the diagnosis of recurrence. An increase in CA125 levels to twice the postoperative nadir was considered as "doubling" at any time during follow up.

**Results:**

Analysis is based on 56 patients who completed primary treatment and who presented for a total of 274 follow-up episodes. Of those, 29 patients (52%) developed a recurrence within the follow up period. Recurrence was diagnosed by CA125 alone in 14 of 29 patients (48%). CA125 was not elevated in 7 patients (24%) who recurred. Doubling of CA125 from nadir was observed in 27/29 patients. Of those 27 patients the doubling from nadir occurred within the normal range of 35 U/ml in 3 cases and outside the normal range in 24 cases. Multivariate analysis suggests that doubling of serum CA125 (OR 5.10, p 0.036) and nadir CA125 > 10 U/ml (OR 2.86, p 0.01) remained the only independent factors to predict ovarian cancer recurrence.

**Conclusions:**

The present paper proposes the validation of a novel CA125 algorithm aiming to detect recurrent EOC. These data may allow us to investigate novel ways of follow up that do not require a patient's physical attendance at a clinic (virtual follow-up).

## Background

World-wide more than 200,000 women are diagnosed with ovarian cancer annually [1]. Due to the lack of effective screening methods, the majority of patients are diagnosed at stages 3 or 4, thus implying poor prognosis [2]. Standard treatment includes a combination of surgery plus chemotherapy and once primary treatment is completed patients are followed regularly. Recurrences following primary treatment are common. The National Comprehensive Cancer Network (NCCN) guidelines suggest that patients are seen every two to four months for two years, every three to six months for another three years and annually thereafter [3]. At every visit the guidelines recommend a physical examination including a pelvic exam and a tumour marker test if the marker was elevated prior to initial treatment. Medical imaging and other blood tests are recommended only when clinically indicated.

In contrast to worldwide current clinical practice of seeing patients for regular follow up for many years, the evidence to support follow up of ovarian cancer patients is sparse [4,5]. Currently, patients are seen face-to-face and have physical examinations as well as a brief history and blood tests as well as imaging regularly. If patients would not need to attend a follow up visit physically, they could be managed through new, virtual strategies. Blood tests, medical imaging and a patient's history could be taken remotely without the need of the patient's physical presence.

CA125 has an established role as a serum tumour marker to triage patients with pelvic masses and has been advocated to predict chemotherapy response in advanced ovarian cancer [6]. Recently we suggested that preoperative serum CA125 is a marker to predict survival in surgical stage 1 EOC [7] and in ovarian tumours of low malignant potential (borderline tumours) (Tang et al., in press, Gynecologic Oncology). Finally, serum CA125 has a long established role in the follow up of patients with EOC [8].

The primary objective of the present study was to evaluate the usefulness of routine serum CA125 levels in the detection of ovarian cancer recurrence.

## Methods

This retrospective case study was carried out at a tertiary gynaecological cancer centre in Queensland, Australia. All the participating patients had surgery with one surgeon (AO) who is a certified gynaecological oncologist (CGO) accredited through the Royal Australian and New Zealand College for Obstetrics and Gynaecology. All patients had provided consent for their data to be used for research purposes. Consecutive patients with all cell types of invasive epithelial ovarian cancer (EOC) treated between 2003 and 2010 were considered eligible (n = 127). Patients with tumours of low malignant potential and with non-epithelial ovarian cancer were not considered eligible. We excluded patients whose aim of treatment was palliative (n = 10) rather than curative or because of a lack of follow-up (n = 39). Patients with no pre-operative CA125 levels (n = 3) or those with pre-operative CA125 levels < 35 U/mL were excluded (n = 18). Patients were eligible to be included if their pre-treatment CA125 levels were elevated (≥ 35 U/mL). IRB approval was not required for this retrospective study.

Following primary treatment, patients were seen every 3 months for three years and 6-monthly for another two years. At each follow-up visit a brief history of symptoms, a physical examination and a serum CA125 level have been obtained. Physical examination included palpation for enlarged groin and supra-clavicular lymph nodes, external abdominal examination and a pelvic examination, which consisted of a speculum visual examination, bimanual vaginal examination, and a rectal examination. Medical imaging was not performed regularly but was only performed when clinically indicated.

Statistical methods: Descriptive statistics were used to present baseline characteristics. Our primary outcome was disease-free survival and was calculated from the date of commencement of initial treatment to the date of recurrence. For the aim of this analysis, patients were censored at the date of their last follow up visit or on the date when recurrence was diagnosed. Variables evaluated as predictors of recurrence include age, stage of the disease, grade, residual disease, cell type, nadir levels of CA125 (categorised as ≤ 10 and > 10) and CA125 levels at follow-up. All variables that showed a p-value < 0.1 on univariate analyses were included in Cox proportional hazards models. CA125 levels were evaluated as a categorical variable coded as 0 if there was no doubling of CA125 and coded as '1' if CA125 levels doubled over the period of follow-up. Stata for windows (version 11.2) was used for statistical analyses.

We recorded if symptoms, findings from physical examination, imaging or serum CA125 levels led to the diagnosis of recurrence. Recurrence has been confirmed histologically or cytologically whenever possible through a fine needle aspirate. In 2 patients recurrence was diagnosed through a laparoscopic investigative procedure and was confirmed histologically. A systematic second look operation was not performed. If histological confirmation of recurrence was not possible, the diagnosis of recurrence was made by imaging. An increase in CA125 levels to twice the postoperative nadir was considered as "doubling" at any time during follow up [9]. If a serum CA125 increased to less than double the nadir, it was repeated in 4 to 8 weeks.

## Results

Analysis is based on 56 patients who had primary surgery and completed chemotherapy as part of their primary treatment and who presented for a total of 274 follow-up episodes. Median follow up was 28.9 months (95% CI: 18-67.2).

Patients' baseline characteristics are shown in Table 1. In brief, 45 patients (80%) had stage 3 or 4 disease and 29 patients (52%) developed a recurrence within the follow up period. Recurrence was diagnosed by serial CA125 measurements alone in 14 of 29 patients (48%). A diagnosis of recurrence was established in 7 patients (24%) when CA125 was not involved.

**Table 1:** Baseline characteristics of Patients included in this study

Characteristic	Values
Age, mean (SD) [years]	62.2 (10.8)
Pre-op CA125, median (IQR)	305 (136-894)
FIGO stages (3 or 4), n (%)	45 (80.4)
No residual disease, n (%)	29 (51.8)
Surgical procedures, n (%)	
Salpingo oophorectomy	54 (96.4)
Omentectomy	49 (87.5)
Pelvic Lymph node dissection	45 (80.4)
Hysterectomy	41 (73.2)
Aortic Lymph node dissection	39 (69.6)
Appendicectomy	26 (46.4)
Rectal resection	13 (23.2)
Liver/Spleen resection	5 (8.9)
Vaginectomy	5 (8.9)
Small bowel resection	4 (7.1)
Diaphragm	3 (5.4)
Ureter	3 (5.4)
Others*	8 (14.3)
Cell type	
Serous, n (%)	36 (64.3)
Other, n (%)	20 (35.7)
Grade, n (%)	
1	4 (7.1)
2	14 (25.0)
3	38 (67.9)
Relapse, n (%)	29 (51.8)
Relapse, detected by, n (%)	
CA125 alone	14 (48.3)
CA125 plus any other (imaging, clinical)	8 (27.6)
CA125 not elevated	7 (24.1)

*Abdominal wall resection; thoracoscopic surgery; hernia repair; distal pancreatectomy; hemicolectomy; large bowel resection.

Of the 29 patients with recurrence, a doubling in CA125 from nadir was observed in 27 patients, whereas in two patients CA125 did not double. Of those 27 patients the doubling from nadir occurred within the normal range of 35 U/ml in 3 cases and outside the normal range in 24 cases. In one additional patient the CA125 increased from 11 U/ml to 21 U/ml and she was also diagnosed with recurrence thereafter.

Of all 56 patients, a doubling from nadir was observed in 37 patients. From those 37 patients, doubling outside the normal range of 35 U/ml occurred in 25 patients and all of these patients were diagnosed with recurrence. Doubling from nadir within the normal range (≤ 35 U/ml) occurred in 12 patients and three of those patients were diagnosed with recurrence subsequently.

Doubling of CA125, advanced stage of the disease, serous cell type, and a nadir CA125 levels higher than 10 U/ml showed significant associations with recurrence in univariate analysis (Table 2). Of these covariates, only a nadir CA125 levels higher than 10 U/ml and doubling of CA125 predicted recurrences in multivariate models (Table 3).

**Table 2:** Univariate association of prognostic factors with recurrence

Variable	HR	95% CI		P
Age	0.97	0.94	1.01	0.150
CA125 doubling	6.19	1.47	26.12	0.013
CA125 nadir > 10 U/ml	2.92	1.36	6.27	0.006
FIGO stages 3 or 4	5.50	1.30	23.30	0.021
Cell type (serous vs. other)	2.79	1.13	6.89	0.026
Grade 1	Referent			
Grade 2 vs. 1	0.60	0.12	3.13	0.544
Grade 3 vs. 1	1.25	0.29	5.38	0.763
Residual disease (any vs. nil)	1.93	0.92	4.05	0.083
Nadir levels > 10 U/ml	2.92	1.36	6.27	0.006

**Table 3:** Multivariate Cox proportional hazards model predicting recurrence

Variables	HR	95% CI		P
CA125 doubling	5.10	1.11	23.43	0.036
FIGO stages 3 or 4 vs stage 1 or 2	1.52	0.28	8.30	0.629
Nadir CA125 levels > 10 U/ml	2.86	1.29	6.37	0.010
Residual disease (any vs. nil)	1.06	0.48	2.33	0.893
Cell type (serous vs. other)	1.52	0.55	4.15	0.417

## Discussion

Our study supports the hypothesis that doubling of serum CA125 and a nadir CA125 > 10 U/ml after completion of first line treatment are associated with a significantly increased risk of recurrence. Patients with EOC whose serum CA125 was elevated but dropped to normal levels after initial treatment and who experienced a doubling of CA125 relative to her nadir, are at very high risk of developing a recurrence.

Current NCCN guidelines for the follow up of patients with EOC are widely accepted but only limited evidence is available to support current clinical practice [3]. There are no clinical trials available to determine optimal follow up intervals, sequence or duration of follow up.

A limited number of retrospective publications address the issue of the method by which ovarian cancer recurrence is detected. A series of 80 patients from Hong Kong suggested that 42 patients (52.5%) had positive findings on physical examination. However, in none of those, positive physical examination finding was the first sign of recurrence. Overall, 73 patients (91%) had raised CA125 at the time of recurrence[4]. A series from Israel including 69 patients with 43 recurrences suggested that abnormal findings from physical examination yielded a sensitivity rate of only 34.9% [5]. The diagnosis of recurrence was based on an abnormal physical examination alone in only 2 (4.6%) patients. Both studies focussed on the value of physical examination in the follow up for EOC. A study by Fehm et al. suggested that CA125 had the highest sensitivity to detect ovarian cancer recurrence, followed by medical imaging and physical examination [10]. In an Italian series including 331 asymptomatic ovarian cancer patients with recurrent EOC, CA125 alone or in combination with medical imaging detected 58% of all recurrences [11]. More recent studies explore the value of serial PET-CT examinations for the detection of recurrent EOC but PET-CT may not be available to the majority of women or to women in non-metropolitan areas.

In three of the above studies, CA125 was considered elevated if it exceeded the upper limit of normal [4,5,10]. The Italian series considered an elevated CA125 if CA125 doubled from the upper limit of normal or if there was a doubling of CA125 from the nadir if CA125 levels were persistently elevated [11]. A study by Crawford et al. using data from 79 patients, showed that CA125 within normal ranges carries prognostic information for survival outcomes [12]. Similarly, the prognostic role of CA125 has been shown in multicentric studies [13,14].

The present series suggests that a doubling of serum CA125 from its nadir is highly and strongly predictive for recurrence even if the CA125 level is still within the upper limit of normal. Our series included 29 recurrences and in 27/29 patients a doubling of CA125 from nadir was observed. Three of these patients doubled their CA125 within the normal range of CA125 (≤ 35 U/ml). Another patient diagnosed with recurrence increased her CA125 1.9-fold (from 11 U/ml to 21 U/ml) and missed the criteria of doubling. Our data suggest that extending the criteria of "doubled CA125 even within the normal CA125 range" might potentially increase the detection of recurrences by 11% to 15%.

While health care professionals and patients agree that follow up is important, their expectations differ. Patients accept inconvenience, costs and physical discomfort associated with regular physical examinations hoping that recurrent disease is detected at an early stage when the tumour load is small and still amenable for curative treatment [15,16]. In contrast, clinicians feel that physical examinations are unlikely to result in survival benefits for patients [8]. Health care professionals' expectations to follow up include the monitoring of symptoms associated with previous treatment or relapse [15,16] and quality assurance.

Potentially, these findings could have an impact on the service provision for ovarian cancer patients. While evidence suggests that patients with ovarian cancer benefit from specialised gynaecological oncology input for reasons mentioned above, a significant percentage of ovarian cancer patients are still not referred to specialised services in large parts of the world. Providing virtual follow up to patients might overcome a geographical barrier, make follow up services available and attractive to patients in non-metropolitan areas at low cost and make management by gynaecological oncology specialist more attractive.

Given the small sample size, we acknowledge that this study is only hypothesis-generating. We plan to extend our sample size and in order to validate these findings.

## Conclusions

In contrast to poor evidence form retrospective or observational studies, consensus exists amongst both, patients and health care providers that follow up is important. The present paper proposes the validation of a novel CA125 algorithm aiming to detect recurrent EOC. These data may allow us to investigate novel ways of follow up that do not require a patient's physical attendance at a clinic.

## Competing interests

The authors declare that they have no competing interests.

## Authors' contributions

EV and AO conceived this study. AO and SKC designed this study. SKC performed the statistical analyses and all the authors interpreted the results. All the authors contributed to draft this manuscript. All authors have read and approved the final draft of the manuscript.
